# Effect of Grain Structure of Gold Plating Layer on Environmental Reliability of Sintered Ag-Au Joints

**DOI:** 10.3390/ma17081844

**Published:** 2024-04-17

**Authors:** Youshuo Ma, Xin Li, Hongyu Zhang

**Affiliations:** 1 School of Materials Science and Engineering, Tianjin University, Tianjin 300350, China; 2021208128@tju.edu.cn (Y.M.); 2018208241@tju.edu.cn (H.Z.); 2 Tianjin University Binhai Industrial Research Institute Co., Ltd., Tianjin 300350, China

**Keywords:** sintered silver joint, gold plating layer, reliability, high thermal storage, hygrothermal aging

## Abstract

Gold-plated substrate is widely used in sintering with silver paste because of its high conductivity, stability, and corrosion resistance. However, due to massive interdiffusion between Ag and Au atoms, it is challenging for sintered Ag-Au joints to maintain high reliability. In order to study the effect of grain structure of gold plating layer on the environmental reliability of sintered Ag-Au joints, we prepared four substrates with different gold structures. In addition to the original gold structure (Au substrate), other gold structures were obtained by heat treatment at temperatures of 150 °C (Au-150 substrate), 250 °C (Au-250 substrate), and 350 °C (Au-350 substrate) for 1 h. Compared with the other three gold substrates, the sinter jointed on the Au-350 substrate obtained the highest shear strength. By analyzing the grain structure of the gold plating layer, it is found that the average grain size of the Au-350 substrate is the largest, and the proportion of low-angle grain boundaries is less. Few grain boundaries have a positive impact on inhibiting the excessive diffusion of Ag atoms and improving the bonding performance of the joint. Based on the above study, we further evaluated the environmental reliability of sintered joints. In 150 °C high-thermal storage, the interdiffusion of Ag and Au in the sintered joint on the Au-350 substrate was restricted, retaining stronger bonding until 200 h. In a hygrothermal environment of 85 °C/85% RH, the shear strength of the sintered Ag-Au joint with the Au-350 substrate maintained above 40.2 MPa during 100 h aging. The results indicated that the sintered Ag-Au joint on the Au-350 substrate with the largest grain size has superior high thermal reliability and hygrothermal reliability.

## 1. Introduction

The third-generation wide-bandgap semiconductor materials are widely used in power devices because of their high energy conversion rate [1,2]. These power devices will generate a lot of heat, resulting in poor reliability. But the traditional lead-free solder has a low melting point and is prone to form intermetallic compounds [3,4,5]. There is an emerging requirement for die-attach material that should be applied above 500 °C [6]. Owing to superior thermal and electrical conductivity and high melting point, silver paste has become the most interesting choice for a new type of attachment material [7,8]. In the view that particle surface energy and interface energy determine the sintering driving force, much research proposed that mixing nano-Ag particles into micro-Ag paste is an available way to reduce auxiliary pressure and sintering temperature [8,9]. To avoid the agglomeration of Ag particles at low temperatures, it is necessary to add organic solvent in silver paste which can remove in sintering [10,11].

During the sintering process, electroplating Ag or Ni/Au is usually used to prevent oxidation of the copper substrate. At the same time, Au and Ag are also commonly used metallization layers that interdiffused with sintered silver during the sintering process to strengthen the connection between the chip and the substrate [12]. Owing to excellent corrosion resistance and weldability, gold surface finish holds the potential to realize a strong joint with silver paste [13,14]. Muralidharan et al. [15] held the view that Au plating promoted the sintered Ag layer densification. Wang et al. [16] prepared different gold structures by using electroless plating and electroplating processes and concluded that the bonding performance of the sintered Ag-Au joints depends on the grain structure of gold surface finish. Zhang et al. [17] obtained different gold structures through different heat treatment processes. The study found the shear strength of sintered silver joints on gold-plated substrates after preheating increased from 13.8 MPa to 25.4 MPa with preheat temperature increasing, and proposed that preheat treatment can improve the joint quality by coarsening grain on gold surface finish.

Because power devices must serve in harsh environments long term, environmental reliability such as thermal stability of sintered Ag layer on gold-plated substrates has received a great deal of attention [18]. Chen et al. [19] conducted thermal cycling tests on sintered Ag-Au joints in the range of −50~250 °C and found that with Au atoms gradually diffusing into the sintered silver layer, the delamination near the Ag-Au interface formed, leading to the shear strength of joints decreasing significantly. Chen and Zhang et al. [20] carried out a 250 °C high thermal aging test on the sintered Ag-Au joints on electroplated Ag and sputtered Ag metallization substrates and revealed that the grain size and orientation of the top metallization Ag layer influence the bonding reliability. Since the grain structure can change the bonding performance of the joint, it is of great significance to study the effect of grain structure of the gold plating layer on the reliability of Ag-Au joints.

Kovar alloy is a fixed expansion alloy with high strength/hardness and corrosion/wear resistance. It is easy to process and has good weldability. It is widely used in aerospace, national defense, and other fields. Compared with traditional direct bond copper (DBC), Kovar alloy has a linear expansion coefficient closer to Si, which can effectively reduce the thermal stress of the chip-substrate sintered silver layer and improve the reliability. It is commonly used in chip substrates, bases, and lead frames for semiconductor device packaging; however, its thermal conductivity is poor, which limits its application in high temperature conditions [21,22]. Lu et al. [21] used electroless plating to prepare Fe and Cu layers on the surface of Kovar alloy powder, and hot-pressing sintered the Cu/Fe/Kovar alloy composite to improve the thermal conductivity of Kovar alloy. Considering the thermal and electrical conductivity between the chip and the substrate under harsh working conditions, a gold plating layer is needed to avoid oxidation, increase conductivity, and reduce thermal resistance. In the realms of national defense and aerospace, the paramount consideration for material application is the assurance of safety and reliability. Although the production cost of Kovar alloy and gold materials is relatively high, their application value cannot be estimated by cost.

In this study, we investigated the effect of a gold-plated layer on the reliability of the sintered joints based on the application background of gold-plated Kovar alloy. Four kinds of gold-plated substrates with different Au grain structures were prepared by different preheat treatments, and their grain structures were observed by electron backscattered diffraction (EBSD). Moreover, the high-temperature reliability at 150 °C and 85 °C/85% RH hygrothermal reliability of sintered Ag-Au joints were evaluated, respectively. It is of great significance to clarify the relationship between interfacial microstructures and environmental reliability of the sintered joint.

## 2. Experiment

**Figure 1:**
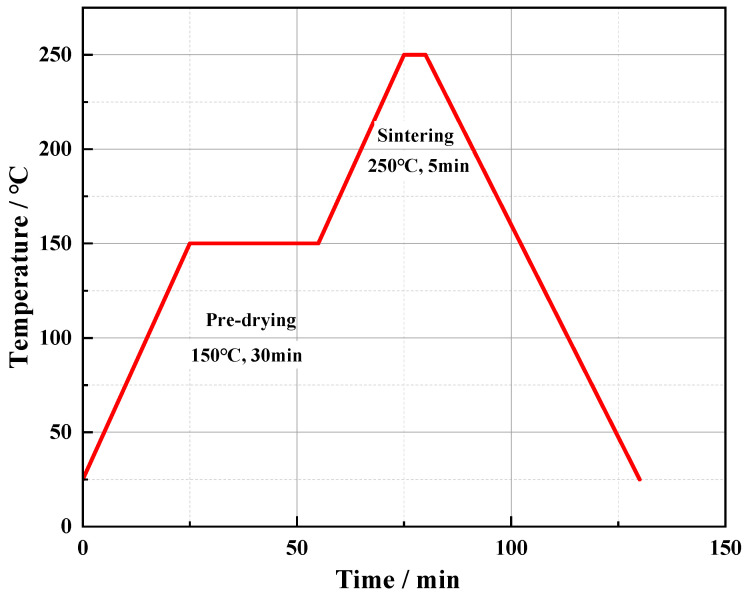
Sintering process.

The substrate we use in this article is Kovar alloy electroplated with Ni (4 μm) and Au (1.3 μm). Based on the influence of temperature on gold structure obtained in preliminary experiments, this article uses heat treatment temperatures of 150 °C, 250 °C, and 350 °C for 1 h to obtain different gold structures. To simplify, four kinds of gold-plated substrates, including the substrate without preheating, are referred to as Au substrate, Au-150 substrate, Au-250 substrate, and Au-350 substrate. The silver paste was stencil-printed with a thickness of 50 μm on the substrate, and then the Si chip (3 × 3 × 0.2 mm^3^) was placed on. The process of sintering is shown in Figure 1. Based on the process research of the sintered silver paste used in this article, it was predried at 150 °C for 30 min, and then heated to 250 °C for 5 min. This is because the diffusion rate of Ag in Au is greater than the diffusion rate of Ag in Ag when the temperature exceeds 150 °C. The purpose of holding at 150 °C for 30 min is to remove the solvent in the paste with few diffusions of Ag in Au. High-temperature (250 °C) and short-time (5 min) sintering enables surface diffusion between Ag atoms to complete densification sintering, reducing the “non-densification” diffusion of Ag at the Ag-Au interconnect interface. The application of this process will make a certain contribution to the improvement of the quality of Ag-Au interconnect joints. At least three assembled samples of each type of joints were tested by the shear tester (Condor 150, XYZTEC, Panningen, The Netherlands) to obtain an average shear strength. To clarify the differences in the grain structures on gold plating layers, the electron backscattered diffraction (EBSD) technique with Orientation Imaging Microscopy 7.2 (OIM 7.2) measure software was used to characterize the grain size distribution and grain boundary misorientation. The cross-sectional microstructures of these samples were observed by the scanning electron microscope (SEM, Hitachi-S4800, Hitachi, Ltd., Tokyo, Japan), and the porosity and pore size of sintered layers were obtained. In addition, the sintered Ag-Au joints were aged at 150 °C and 85 °C/85% RH, respectively. According to microstructure evolution and strength changes of sintered joints, the reliability was evaluated and compared.

## 3. Result and Discussion

**Figure 2:**
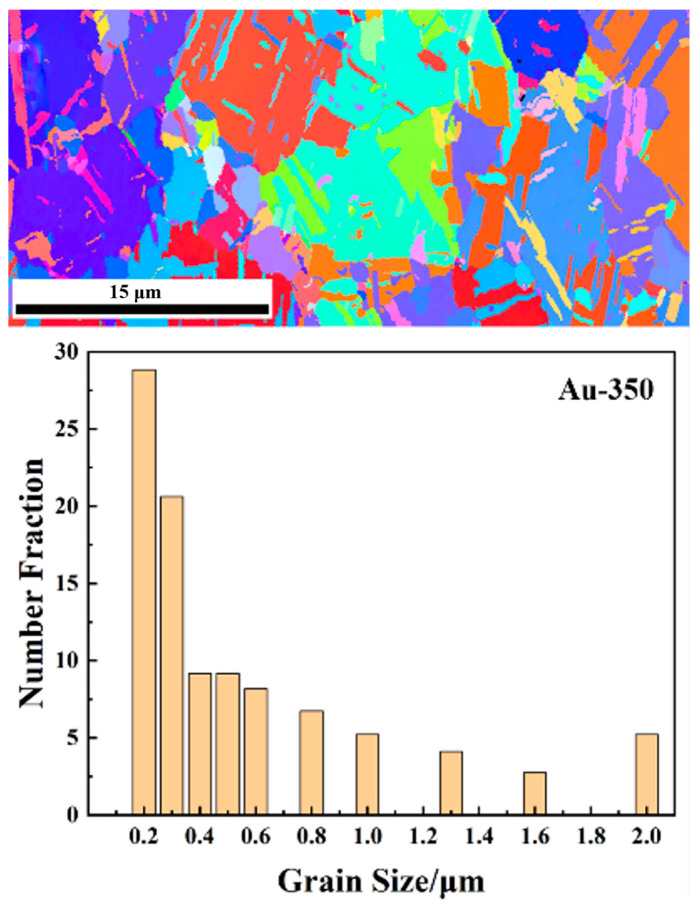
EBSD image and grain size distribution of the gold plating layer on the Au-350 substrate, grain orientation represented by different colors.

The shear strength of sintered Ag-Au joints on Au, Au-150, and Au-250 substrates, and the Au grain size distribution and the grain boundary orientation difference of Au, Au-150, and Au-250 substrates were given in the previous study [23] of the authors of this article. The average shear strength of the sintered Ag-Au joint on the Au-350 substrate was the highest amongst four kinds of Ag-Au joints, reaching 46.1 MPa, which is 39.72% higher than that on the Au substrate. The EBSD image and grain size distribution of gold plating layer on the Au-350 substrate is shown in Figure 2. The average grain of the Au-350 substrate was 0.56 μm. The grains of the Au-350 substrate coarsened to 10 times that of the substrate without preheating. Referring to a misorientation of 10° is commonly used in the distinction of the type of grain boundaries; in this study, the grain boundary misorientation less than 10° is called LAGB (low-angle grain boundary). The proportion of LAGBs of the Au-350 substrate was 3.80%. As shown in Figure 2, there are larger grains with more high-angle grain boundaries on the gold-plated layer on the Au-350 substrate. It is interesting to note that the grain size and the type of grain boundary work together to improve the bonding strength of the sintered Ag-Au joint on the Au-350 substrate.

### 3.1. High-Temperature Reliability of Sintered Ag-Au Joints

**Figure 3:**
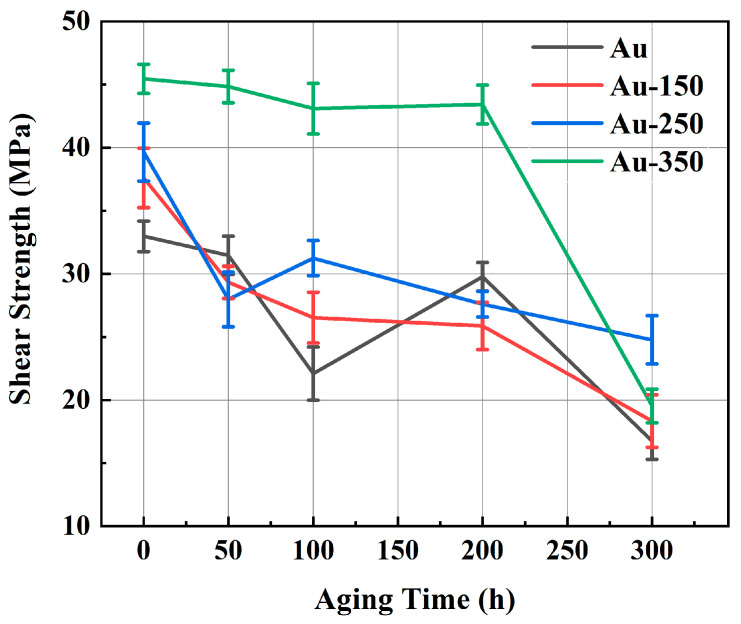
Shear strength of four kinds of Ag-Au joints with high-temperature aging.

As a control group before aging, the shear strength of sintered Ag-Au joints on Au, Au-150, and Au-250 substrates was obtained in the previous study [23] of the authors of this article. Figure 3 depicts the shear strength of sintered Ag-Au joints on substrates with different gold plating layers during 300 h of thermal aging at 150 °C. As shown in Figure 3, for the Au substrate, the shear strength of the Ag-Au joint fluctuated dramatically, decreasing to half of the initial strength after 300 h aging. The shear strength of joints sintered on the Au-150 substrate decreased slowly but continuously. For the Au-250 substrate, the bonding strength of sintered joint decreased rapidly at the initial stage of aging, but the decreasing speed became slower with aging time. Different to other gold-plated substrates, the sintered Ag-Au joint on the Au-350 substrate remained above 43.0 MPa after aging for 200 h, which is 59.25% higher than that of the sintered Ag-Au joint of the Au substrate, and the strength fluctuation ranged within only 5 MPa until 200 h. It can be seen that the sintered Ag-Au joint on the Au-350 substrate shows superior high thermal reliability.

**Figure 4:**
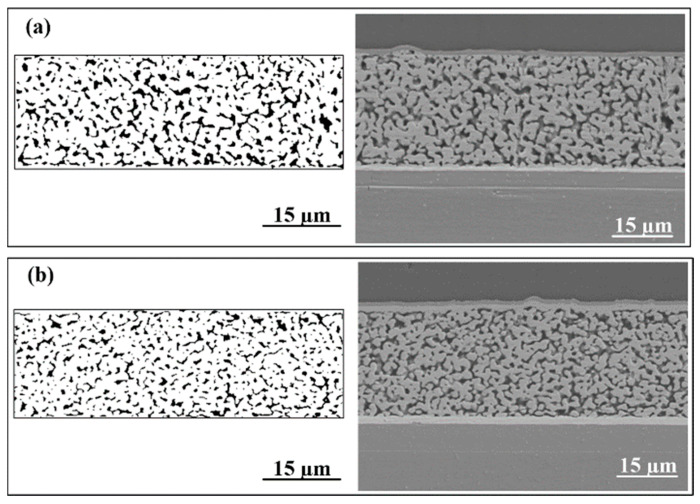
Microstructure of Ag-Au joints on different substrates after 200 h high temperature aging: (**a**) Au substrate and (**b**) Au-350 substrate.

In order to further explain the influence of the grain structure of the gold plating layer on the bonding quality and explore the fracture modes, the cross-section samples of the sintered Ag-Au joint on the Au substrate and Au-350 substrate after aging at 150 °C for 200 h were observed, as shown in Figure 4.

The percentage of pore area in SEM images can be quantified by MATLAB based on the light reflectivity on the cross-section of the joint. After aging for 200 h, the porosity of the Ag-Au joint sintered on the Au substrate was approximately 17.13%. On the contrary, the Ag-Au joint sintered on the Au-350 substrate showed good contact, and pores of the sintered silver layer are refined and the porosity of sintered silver layer is just 13.43%.

The diffusion flux of Ag atoms refers to the amount of Ag atoms per unit area per unit time perpendicular to the diffusion direction. The grain size of the Au-350 substrate was large, resulting in insufficient Ag-Au interdiffusion as sintered; however, the high-temperature test promoted the diffusion flux of silver atoms increasing by extending high thermal hold time, which makes the Ag-Au connection stronger and contributes to excellent shear strength.

**Figure 5:**
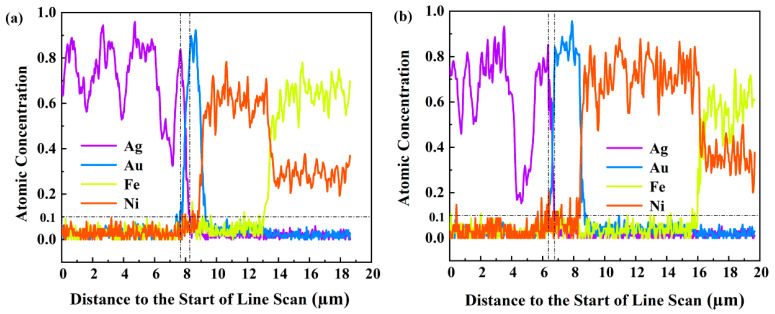
Diffusion zones of the joint interface between sintered Ag-Au joints on different substrates after 200 h high-temperature aging: (**a**) Au substrate and (**b**) Au-350 substrate.

The technique of EDS (energy dispersive spectroscopy) line scanning was used to monitor the diffusion of the Ag-Au interface. Figure 5 shows the line-scanning results in the interfacial diffusion zones of two kinds of Ag-Au joints after aging for 200 h. The atoms percentages of the two kinds of atoms at different positions were normalized, and the dividing lines show that the concentration of Ag and Au atoms reduced to 10% of their original concentration, respectively. The area covered by two dividing lines of the Ag and Au element curve is defined as the Ag-Au interdiffusion band, and the thickness of the diffusion band can be obtained by measuring the distance between the two dividing lines. According to the measurement result, the Ag-Au interface diffusion bands of sintered joints on the Au substrate and the Au-350 substrate after aging for 200 h were 0.59 μm and 0.27 μm, respectively. Therefore, it is considered that the Ag-Au interdiffusion happened on sintered Ag-Au joints of the Au substrate more severely. In the previous study, the joints formed a thick interdiffusion zone; meanwhile, there was a high porosity layer above the zone, weakening bonding. As a result, the reliability deteriorated when the diffusion band of joints on the Au substrate became thicker. However, the Ag-Au diffusion zone of joints on the Au-350 substrate is thinner, and the joint has superior thermal reliability.

### 3.2. Hygrothermal Reliability

**Figure 6:**
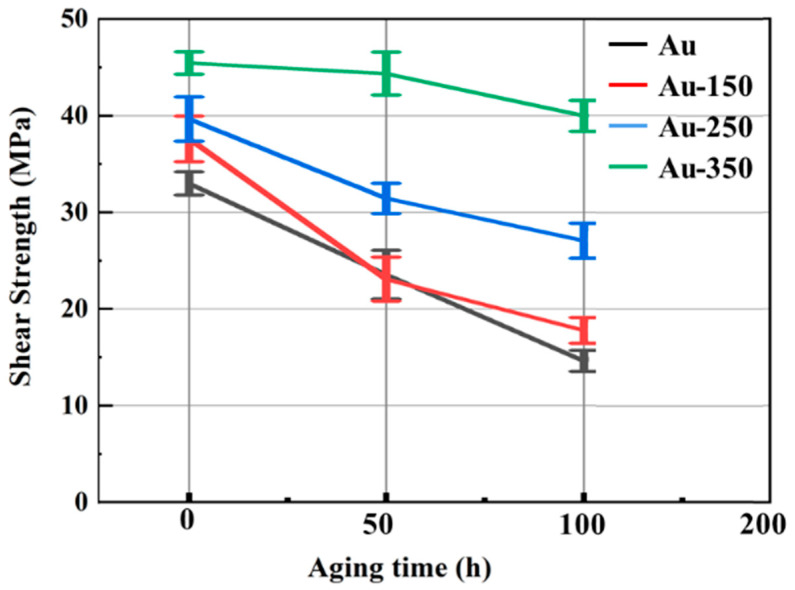
Shear strength of four kinds of Ag-Au joints with hygrothermal aging.

To evaluate the reliability of joints in severe environments, the environment of 85 °C/85% RH was employed to storage the joints. Figure 6 shows the shear strengths of four kinds of Ag-Au joints during aging in a hygrothermal environment.

Until 50 h aging, the trend of shear strength on the Au-150 substrate was almost the same as that of the Au-250 substrate. After 100 h aging, the shear strength of the sintered Ag-Au joint on the Au substrate sharply decreased to 14.6 MPa, which was 55.67% lower than that of its as-sintered joint. However, the shear strength of the Au-350 substrate remained at 40.0 MPa, which was only 12.04% lower than its as-sintered joint.

**Figure 7:**
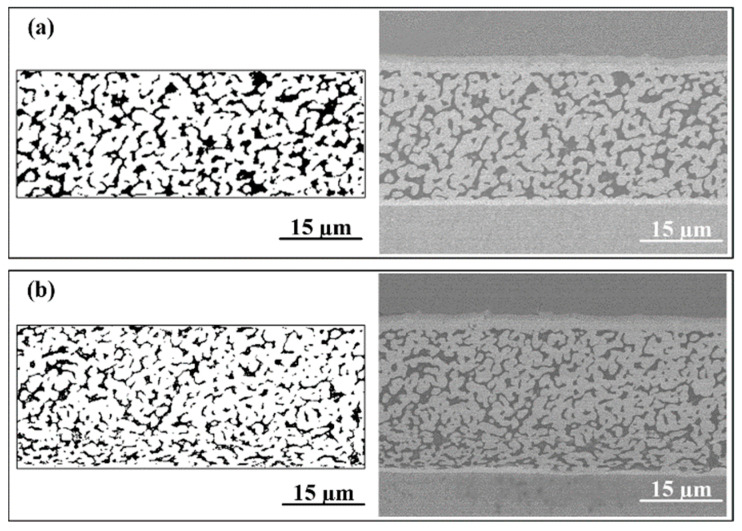
Microstructure of Ag-Au joints on different substrates after 100 h hygrothermal aging: (**a**) Au substrate and (**b**) Au-350 substrate.

Figure 7 shows the cross-sectional morphology of the sintered Ag-Au joint and pore diagram of the sintered silver layer for the Au substrate and the Au-350 substrate after aging in an environment of 85 °C/85% RH for 100 h. As shown in Figure 7a, there were few sintered necks and a lot of large pores in the sintered Ag-Au joint on the Au substrate. The porosity of the sintered silver layer was as high as 21.30%, calculated by MATLAB. As Figure 7b, the sintered silver layer of the Au-350 substrate was relatively denser and more uniform; most of the silver particles are neck-connected with the surrounding particles, and there are few isolated particles. The porosity was calculated to be 16.70%, which was 21.60% lower than that of the Au substrate. Owing to lower porosity and uniform pores, the Ag-Au joint sintered on the Au-350 substrate shows superior hygrothermal reliability.

**Figure 8:**
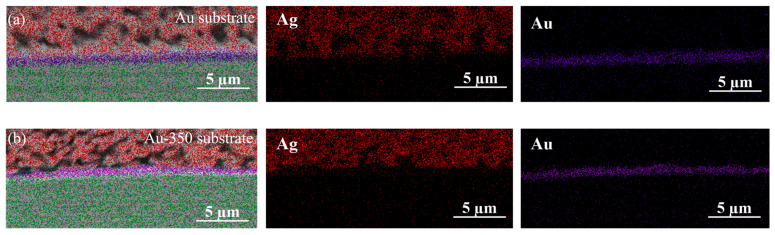
EDS area scan analysis of sintered Ag-Au joints on electrolytic Au under different preheated states after 100 h hygrothermal aging: (**a**) Au substrate and (**b**) Au-350 substrate. Red represents the silver element, purple represents the gold element.

Figure 8 illustrates the element distribution on the Ag-Au interface of two kinds of sintered joints. It is interesting to note that there was a thicker dense layer with weak sintered necks and larger pores above the Ag-Au interface of the sintered joint on the Au substrate. Through measuring the element content of the 1.67 μm silver layers above the interface, it was found that the Ag atom ratio on the Ag-Au joints in the Au substrate was only 29.77% and that of the Au-350 substrate was 46.47%. The depletion of silver atoms above the interface is the main reason for the faster decreased shear strength of the sintered joint on the Au substrate. The finer grains with more diffusion paths on the Au substrate promoted Ag atoms near the interface diffusing into Ag-Au interface rapidly; however, the Ag atoms far away from the interface migrated slowly, resulting in the delamination and weak bonding. Combined with the observation of the densified sinter layer with finer pores on the Au-350 joint, it indicates that the microstructure of substrate after preheating at 350 °C plays a positive part in the density of the bonding layer.

## 4. Conclusions

In this paper, the effects of the grain structure of gold plating layer on the initial bonding properties and thermal and hygrothermal reliability of sintered Ag-Au joints were studied. The bonding strength and microstructure of sintered joints on four kinds of substrates were compared. Finally, we draw the following conclusions:

(1) With preheating temperature increasing, the bonding strength of sintered Ag-Au joints increased. Among the four kinds of joints, the sintered Ag-Au joint on the Au-350 substrate showed the best bonding performance.
(2) Using EBSD characterization, it was found that the weighted average grain size of the Au-350 substrate is the largest, and the proportion of LAGBs accounts for only 3.80%. It is worth noting that HAGBs promote diffusion, and large grains with fewer grain boundaries reduce diffusion pathways on the Au-350 substrate. The two factors work together to inhibit the excessive diffusion of Ag atoms and improve the shear strength of the joint.
(3) During 200 h aging at 150 °C, the shear strength of the sintered joint on the Au-350 substrate fluctuated in the range from 43.0 MPa to 45.2 MPa, which was about 10.0 MPa higher than that on the Au substrate. It indicated that the sintered Ag-Au joint obtained at the Au-350 substrate has excellent high thermal reliability.
(4) The shear strength of the sintered Ag-Au joint on the Au substrate declined abruptly after 100 h aging in the hygrothermal environment of 85 °C/85% RH. The shear strength of the joint on the Au-350 substrate remained above 40.2 MPa, which was much higher than that of the Au substrate. The sintered Ag-Au joint on the Au-350 substrate showed the denser sintered silver layer with smaller pores contributed to superior reliability in the hygrothermal environment.

This article verifies the stability of Ag-Au joints on Au-350 substrates in high-temperature and hygrothermal environments. It is necessary to further extend the aging time or worsen the test conditions to obtain the failure mechanism and lifespan of the Ag-Au joint. In future research, a reliability life prediction model for Ag-Au joints can be established by setting different test conditions. In addition, we will also conduct power-cycle-related research on devices using different gold-plated structure substrates, and establish power cycle failure analysis. Different from the environmental reliability study in this article, the power cycle test gives a life and reliability evaluation that is closer to real application scenarios. These research results can guide the preparation of Au metallization layers with specific structures and maximize the benefits of various materials in actual production.

## Data Availability

Data are contained within the article.
